# Identification of novel gene expression signature in lung adenocarcinoma by using next-generation sequencing data and bioinformatics analysis

**DOI:** 10.18632/oncotarget.21022

**Published:** 2017-09-18

**Authors:** Ya-Ling Hsu, Jen-Yu Hung, Yen-Lung Lee, Feng-Wei Chen, Kuo-Feng Chang, Wei-An Chang, Ying-Ming Tsai, Inn-Wen Chong, Po-Lin Kuo

**Affiliations:** ^1^ Graduate Institute of Medicine, College of Medicine, Kaohsiung Medical University, Kaohsiung, Taiwan; ^2^ School of Medicine, College of Medicine, Kaohsiung Medical University, Kaohsiung, Taiwan; ^3^ Division of Pulmonary and Critical Care Medicine, Department of Internal Medicine, Kaohsiung Medical University Hospital, Kaohsiung, Taiwan; ^4^ Division of Thoracic surgery, Department of Surgery, Kaohsiung Medical University Hospital, Kaohsiung, Taiwan; ^5^ Graduate Institute of Clinical Medicine, College of Medicine, Kaohsiung Medical University, Kaohsiung, Taiwan; ^6^ Welgene Biotech. Inc, Taipei, Taiwan; ^7^ Department of Respiratory Therapy, College of Medicine, Kaohsiung Medical University, Kaohsiung, Taiwan; ^8^ Center for Biomarkers and Biotech Drugs, Kaohsiung Medical University, Kaohsiung, Taiwan; ^9^ Institute of Medical Science and Technology, National Sun Yat-Sen University, Kaohsiung, Taiwan

**Keywords:** next-generation sequencing, bioinformatics, microRNA, messenger RNA, lung adenocarcinoma

## Abstract

Lung adenocarcinoma is one of the leading causes of cancer-related death worldwide. We showed transcriptomic profiles in three pairs of tumors and adjacent non-tumor lung tissues using next-generation sequencing (NGS) to screen protein-coding RNAs and microRNAs. Combined with meta-analysis from the Oncomine and Gene Expression Omnibus (GEO) databases, we identified a representative genetic expression signature in lung adenocarcinoma. There were 9 upregulated genes, and 8 downregulated genes in lung adenocarcinoma. The analysis of the effects from each gene expression on survival outcome indicated that 6 genes (AGR2, SPDEF, CDKN2A, CLDN3, SFN, and PHLDA2) play oncogenic roles, and 7 genes (PDK4, FMO2, CPED1, GNG11, IL33, BTNL9, and FABP4) act as tumor suppressors in lung adenocarcinoma. In addition, we also identified putative genetic interactions, in which there were 5 upregulated microRNAs with specific targets - hsa-miR-183-5p-BTNL9, hsa-miR-33b-5p-CPED1, hsa-miR-429-CPED1, hsa-miR-182-5p-FMO2, and hsa-miR-130b-5p-IL33. These 5 microRNAs have been shown to be associated with tumorigenesis in lung cancer. Our findings suggest that these genetic interactions play important roles in the progression of lung adenocarcinoma. We propose that this molecular change of genetic expression may represent a novel signature in lung adenocarcinoma, which may be developed for diagnostic and therapeutic strategies in the future.

## INTRODUCTION

Lung cancer is one of the leading causes of cancer-related death worldwide [1]. The development and progression of lung cancer has been widely studied. Briefly, the genetic alterations or mutations occurred in a single cell, leading to cellular transformation and thus expansion into a malignant tumor [2]. Non-small cell lung carcinoma (NSCLC) accounts for about 80–85% of all lung cancers [3, 4], of which lung adenocarcinoma (40%) is the most common subtype of NSCLC [5]. Surgery to remove cancer, with or without radiotherapy/chemotherapy, is the standard approach for early stage lung cancer. However, the recurrence of distant metastasis [6, 7] or resistance to therapy [8, 9] often occurs, and such phenomenon is associated with critical genetic alterations involved in various biological mechanisms.

The genetic alterations related to cellular transformation are involved in various biological processes, including transcription [10], DNA repair [11], cell cycle progression [12], apoptosis [13], migration ability [14], and metabolism [15, 16]. In lung cancer, many genes have been identified as oncogenes or tumor suppressor genes, which modulate varieties of molecular functions involved in tumor development and progression [17, 18]. Recently, small RNAs have been found to play important roles in lung cancer. MicroRNAs are a group of small non-coding RNAs containing 20–26 nucleotides, which can regulate gene expression via binding to the 3′ untranslated region (3′UTR) of specific messenger RNAs (mRNAs). This interaction can cause mRNAs’ degradation or translation inhibition [19]. The signaling pathways associated with microRNAs targeting oncogenes or tumor suppressor genes have been reported to be involved in lung cancer progression [20]. Alterations of many microRNAs in chromosome regions associated with various cancers have also been implicated [21].

Next-generation sequencing (NGS) is a powerful method to screen the entire transcriptomic profile, including messenger RNAs or small RNAs [22]. In this study, we attempted to identify the differentially expressed genes and genetic interactions of target gene-microRNA in lung adenocarcinoma combined with systematic analysis, by using bioinformatics tools, including the Oncomine [23], Gene Expression Omnibus (GEO) [24], PrognoScan [25], Kaplan-Meier plotter [26], SurvExpress [27], and miRmap databases [28]. We sought to identify novel gene expression signature and/or genetic interactions in lung adenocarcinoma via systematic bioinformatics analysis. Hopefully, the approach and findings from this study will provide new perspectives on the development of diagnostic and therapeutic strategies for lung adenocarcinoma.

## RESULTS

### Identification of differentially expressed genes as a molecular signature in lung adenocarcinoma

**Figure 1:**
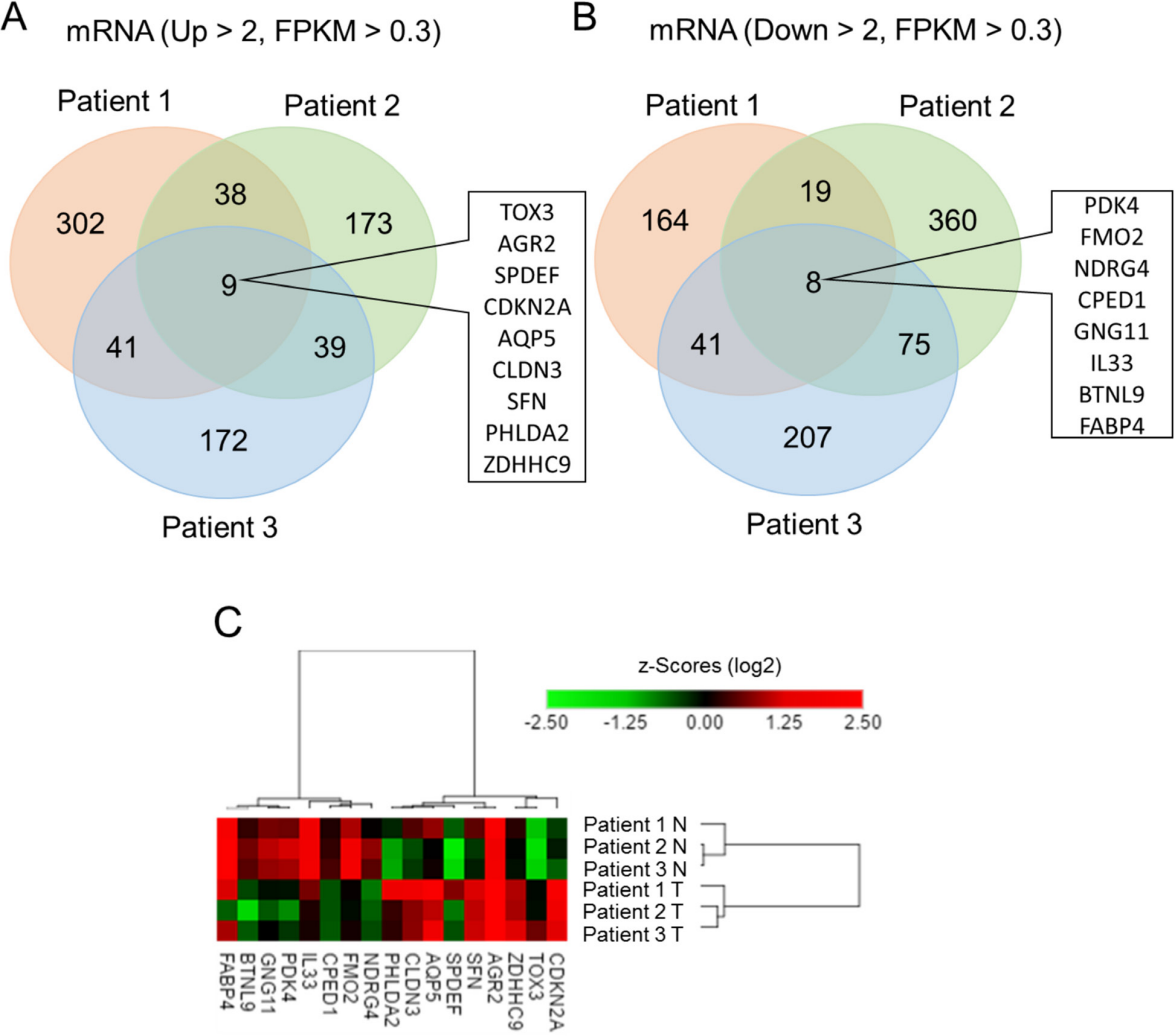
Identification of differentially expressed genes in lung adenocarcinoma compared to adjacent normal tissue using next-generation sequencing Venn diagram analysis showed 9 upregulated genes (**A**) and 8 downregulated genes (**B**) in lung adenocarcinoma, compared to adjacent non-adenocarcinoma tissue from 3 pairs of clinical specimens. The criteria were fold change > 2 (tumor/normal) and fragments per kilobase million (FPKM) > 0.3. (**C**) The heatmap diagram showed the differentially expressed genes with z-score (log2) values by using color clustering on GENE-E web-tool. Green represents downregulation (minimum = −2.5), and red represents upregulation (maximum = 2.5).

**Figure 2:**
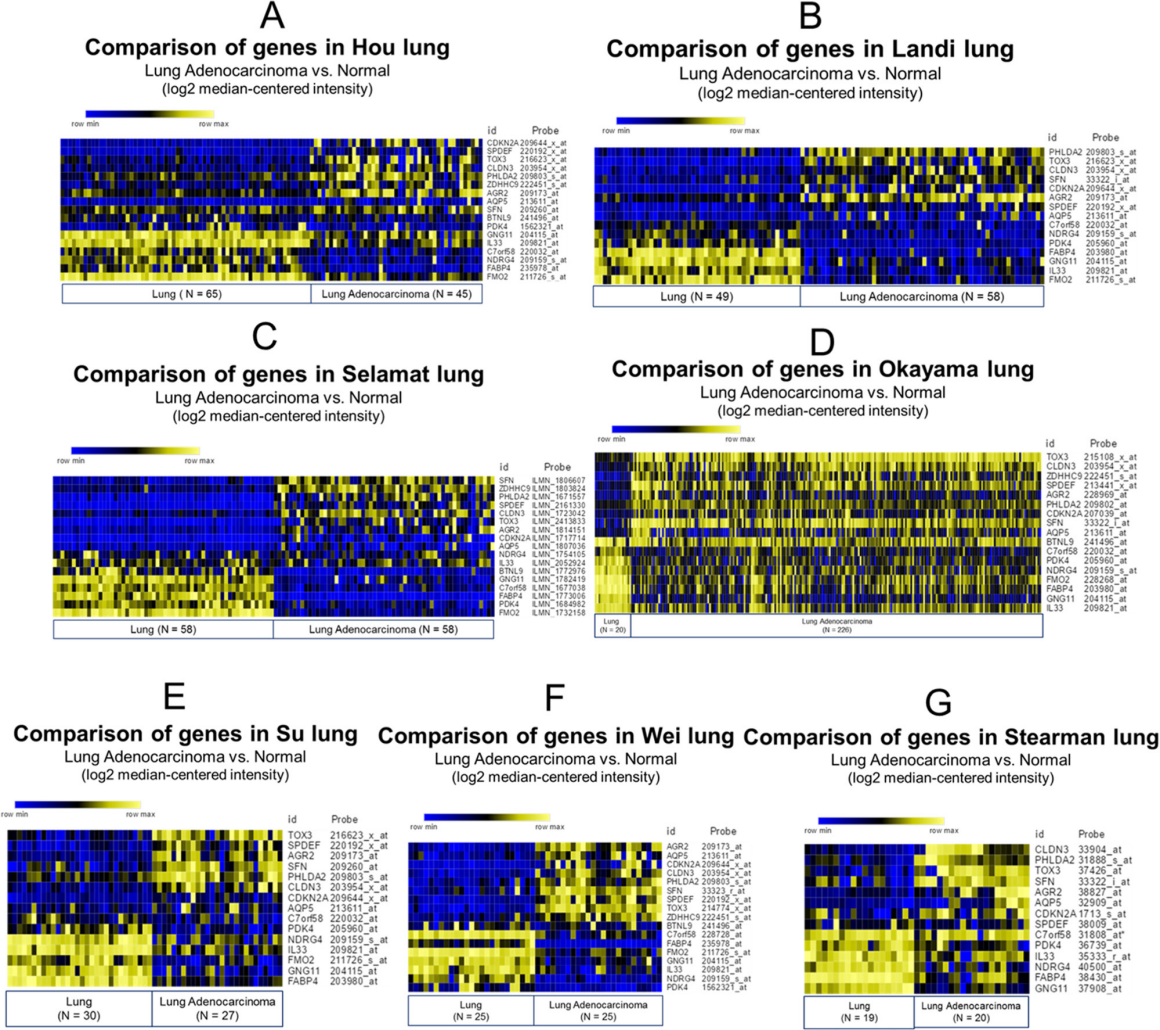
Comparison of differentially expressed genes in clinical lung adenocarcinoma and normal lung tissue by Oncomine database analysis Seven microarray datasets from the Oncomine database were used to analyze gene expression patterns (lung adenocarcinoma vs. normal), including (**A**) Hou, (**B**) Landi, (**C**) Selamat, (**D**) Okayama, (**E**) Su, (**F**) Wei, and (**G**) Stearman. Seventeen differentially expressed genes (9 up and 8 down) identified from 3 pairs of clinical lung adenocarcinoma were selected. Raw data were extracted and re-plotted by GENE-E web-tool, and the relative color scheme used for clustering analysis. Yellow represents high expression (maximum = 1) and blue represents low expression (minimum = 0). The gene symbols and corresponding specific probes are displayed on the right side of each diagram.

To investigate genetic expression changes in lung adenocarcinoma, we analyzed the transcriptomic profiles in 3 pairs of human specimens from lung adenocarcinoma and its adjacent normal lung tissue using next-generation sequencing (Figure 1). We focused on protein-coding RNAs and Venn diagram analysis which showed that 9 genes were upregulated (Figure 1A), whereas 8 genes were downregulated (Figure 1B) in lung adenocarcinoma tissue compared to adjacent normal lung tissue. The analysis criteria were fold change > 2 and fragments per kilobase million (FPKM) > 0.3. The hierarchical color clustering showed the expression pattern of each gene with z-scores (log2) in these 3 pairs of specimens (Figure 1C). The list of 17 differentially expressed genes with FPKM is shown in Table 1. To investigate whether this genetic expression pattern could represent a molecular signature in lung adenocarcinoma, we investigated these genes in the Oncomine database, which contains different data sets of specimens from lung adenocarcinoma and normal lung tissue. We selected 7 datasets from the Oncomine database for comparison of gene expression, including Hou (normal = 65 and lung adenocarcinoma = 45), Landi (normal = 49 and lung adenocarcinoma = 58), Selamat (normal = 58 and lung adenocarcinoma = 58), Okayama (normal = 20 and lung adenocarcinoma = 226), Su (normal = 30 and lung adenocarcinoma = 27), Wei (normal = 25 and lung adenocarcinoma = 25), and Stearman (normal = 19 and lung adenocarcinoma = 20). The heatmap analysis indicated that the genetic expression patterns of 17 genes were similar among these datasets (Figure 2), which suggests that this molecular change is consistent in lung adenocarcinoma, and may represent a novel genetic signature in lung adenocarcinoma.

**Table 1:** Differentially expressed genes identified from next-generation sequencing data

Gene	Description	FPKM ( fragments per kilobase million)						T/N
		N1	T1	N2	T2	N3	T3	
TOX3	TOX High Mobility Group Box Family Member 3	1.65	11.99	0.70	11.57	1.08	31.11	Up
AGR2	Anterior gradient 2, Protein Disulphide Isomerase Family Member	340.96	1530.74	126.15	864.49	148.26	955.51	Up
SPDEF	SAM Pointed Domain Containing ETS Transcription Factor	5.11	37.10	0.48	3.91	0.88	5.90	Up
CDKN2A	Cyclin-Dependent Kinase Inhibitor 2A	7.04	298.07	10.05	129.69	4.94	80.09	Up
AQP5	Aquaporin 5	41.76	333.28	12.70	59.02	14.62	107.83	Up
CLDN3	Claudin 3	24.83	104.71	6.02	38.53	7.31	39.11	Up
SFN	Stratifin	27.16	75.72	7.48	59.63	9.59	65.88	Up
PHLDA2	Pleckstrin Homology-Like Domain, Family A, Member 2	9.07	96.14	2.60	19.93	2.36	18.58	Up
ZDHHC9	Zinc finger, DHHC-type containing 9	17.89	53.14	12.38	49.49	11.08	78.66	Up
PDK4	Pyruvate Dehydrogenase Kinase 4	30.91	10.82	72.24	3.18	47.76	7.63	Down
FMO2	Flavin Containing Monooxygenase 2	50.62	13.47	138.82	11.57	185.08	15.11	Down
NDRG4	NDRG Family Member 4	13.43	3.88	40.80	5.08	26.46	6.07	Down
CPED1	Cadherin-like and PC-esterase Domain Containing 1	16.73	5.70	19.42	5.68	23.24	5.39	Down
GNG11	G Protein Subunit Gamma 11	32.96	11.15	57.85	5.37	42.70	12.85	Down
IL33	Interleukin 33	117.77	33.34	133.98	16.49	153.31	15.49	Down
BTNL9	Butyrophilin-like 9	19.57	6.43	32.15	0.92	26.15	5.25	Down
FABP4	Fatty Acid Binding Protein 4	252.56	74.90	486.96	5.08	459.21	49.26	Down

We classified 17 differentially expressed genes into 6 groups by biological and molecular functions, based on literature searches (Table 2; Supplementary Table 1), including (1) transcription regulation – TOX3 and SPDEF, (2) metabolism – PDK4, FABP4, and FMO2, (3) cell cycle regulation – CDKN2A, PHLDA2, SFN, and NDRG4, (4) cellular migration – AGR2, AQP5, and CLDN3 (5) inflammation – IL33, and (6) undefined group – ZDHHC9, BTNL9, GNG11, and CPED1 (also known as C7orf58). To further elucidate the role of these genetic expression changes in cancer progression, we performed survival curve analysis using the PrognoScan, Kaplan–Meier plotter, and SurvExpress databases.

**Table 2:** Functional classification of differentially expressed genes

Functions	Genes	Fold change (Cancer/Normal)	References^#^
**Transcriptional regulation**	TOX3	UP	1
	SPDEF	UP	2
**Metabolism**	PDK4	DOWN	3
	FABP4	DOWN	4
	FMO2	DOWN	5, 6
**Cell cycle regulation**	CDKN2A	UP	7
	PHLDA2	UP	8, 9
	NDRG4	DOWN	10
	SFN	UP	11
**Cellular migration**	AGR2	UP	12
	AQP5	UP	13
	CLDN3	UP	14
**Inflammation**	IL33	DOWN	15
**Others**	ZDHHC9	UP	16
	BTNL9	DOWN	17
	GNG11	DOWN	18
	CPED1	DOWN	19

^#^ References were list in Supplementray Table 1.

### Analysis of TOX3 and SPDEF in lung adenocarcinoma

**Figure 3:**
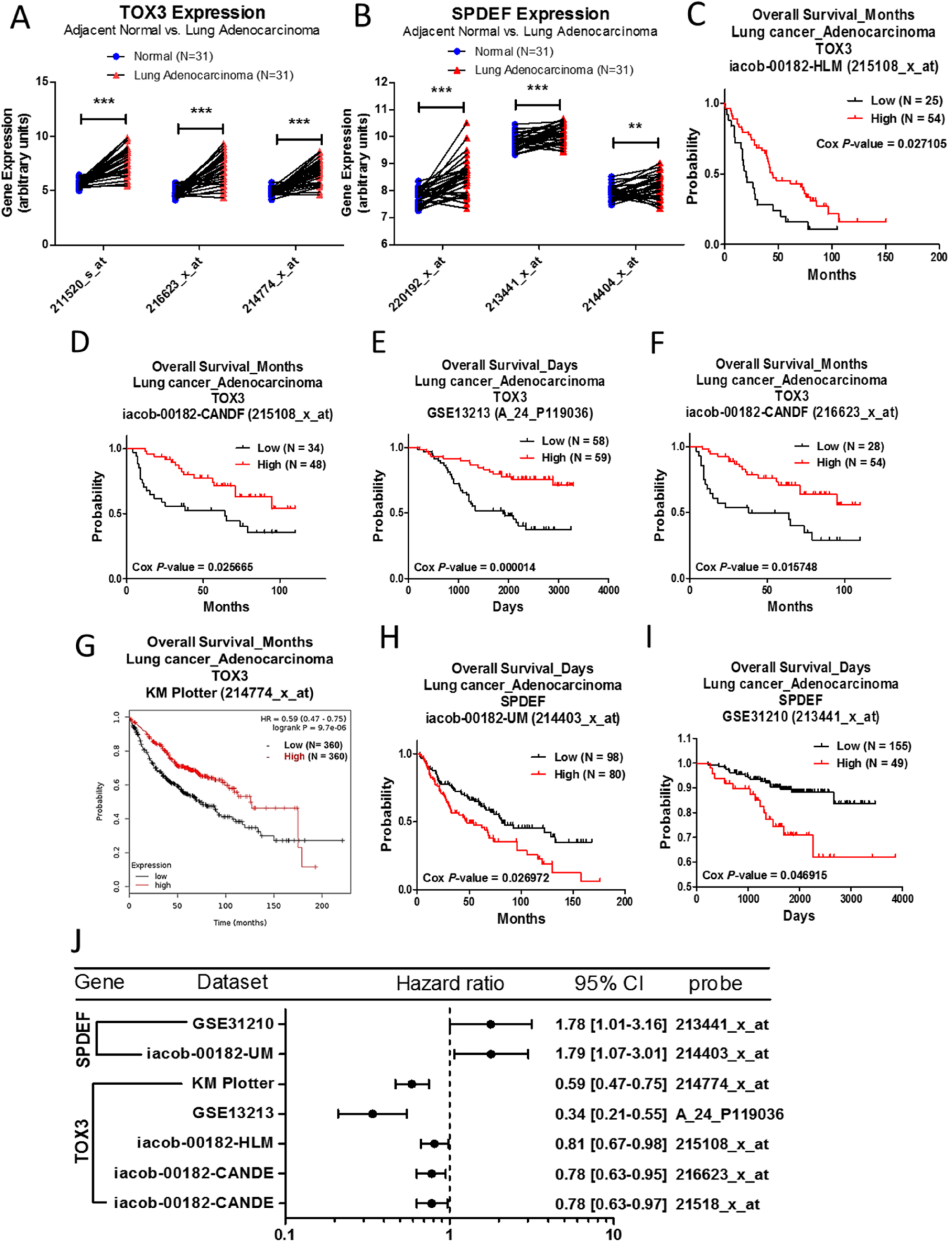
Analysis of TOX3 and SPDEF in clinical lung adenocarcinoma patients using bioinformatics databases The gene expression of TOX3 (**A**) and SPDEF (**B**), comparing 31 pairs of clinical lung adenocarcinoma (red) and adjacent normal tissue (blue), was performed on GSE10072 microarray from the GEO database. (3 probes for TOX3 and SPDEF respectively in GSE10072) The *p*-value of gene expression was calculated by *t*-test with Wilcoxon matched-pairs signed rank test. ^***^ represents *p* < 0.001, ^**^ represents *p* < 0.01. The survival curves comparing 2 populations with high (red) and low (black) gene expression in lung adenocarcinoma patients were performed on the PrognoScan database - TOX3 (**C**–**F**) and SPDEF (**H**, **I**), and the Kaplan–Meier plotter database - TOX3 (**G**). The analysis criteria of the PrognoScan and Kaplan–Meier plotter databases were Cox *p*-value < 0.05 and log-rank *p*-value < 0.05 respectively. The raw data of GEO and PrognoScan databases were extracted and re-plotted by GraphPad Prism 5 software. (**J**) The forest plots showed hazard ratios (95% CI, confidence interval) identified from PrognoScan and Kaplan–Meier plotter databases.

The mRNA expression of TOX3 and SPDEF between lung adenocarcinoma and normal lung tissue derived from the Oncomine database is listed in Table 3. The analysis criteria were fold change > 2, *p*-value < 1E-04, and gene ranking in the top 10%. The results showed that both TOX3 and SPDEF are significantly upregulated in lung adenocarcinoma, compared to normal tissue. In addition, we selected a microarray with the accession number of GSE10072 from the Gene Expression Omnibus (GEO) database for gene expression analysis. This array contains 31 pairs of clinical lung adenocarcinomas and adjacent normal tissue. The results showed that the mRNA expression of either TOX3 (Figure 3A) or SPDEF (Figure 3B) is upregulated in lung adenocarcinoma. To further investigate the role of TOX3 and SPDEF expression in cancer progression, we performed a survival curve analysis to evaluate the effects of gene expression in lung cancer patients with lung adenocarcinoma. The results indicated that the population with higher TOX3 expression has better survival rates (Figure 3C–3G), whereas the population with higher SPDEF expression has poorer survival outcome (Figure 3H, 3I). The prognostic values of TOX3 and SPDEF expression in lung adenocarcinoma were shown as forest plots (Figure 3J), which were derived from the PrognoScan database with a Cox *p*-value < 0.05, and the Kaplan–Meier plotter database with a log-rank *p*-value < 0.05.

**Table 3:** Analysis of TOX3 and SPDEF mRNA expression in lung adenocarcinoma compared to normal tissue from Oncomine database

Gene	Fold change (Cancer/Normal)	*P*-value	Gene Ranking (Top%)	Samples (Normal : Tumor)	Dataset	Probe
**TOX3**	8.685	1.18E-22	1	20 : 226	Okayama	215108_x_at
	19.973	1.86E-24	1	25 : 25	Wei	214774_x_at
	4.968	2.80E-18	2	49 : 58	Landi	216623_x_at
	8.497	1.79E-9	1	30 : 27	Su	216623_x_at
	3.15	3.09E-4	2	17 : 132	Bhattacharjee	37426_at
	12.617	2.17E-7	3	19 : 20	Stearman	37426_at
	3.764	4.72E-7	10	65 : 45	Hou	216623_x_at
**SPDEF**	3.719	1.54E-11	5	20 : 226	Okayama	213441_x_at
	3.451	2.64E-12	1	25 : 25	Wei	220192_x_at
	5.44	2.86E-9	1	30 : 27	Su	220192_x_at
	2.12	1.94E-7	9	65 : 45	Hou	220192_x_at
	3.844	9.6E-17	2	58 : 58	Selamat	ILMN_2161330

### Analysis of PDK4, FMO2, and FABP4 in lung adenocarcinoma

**Figure 4:**
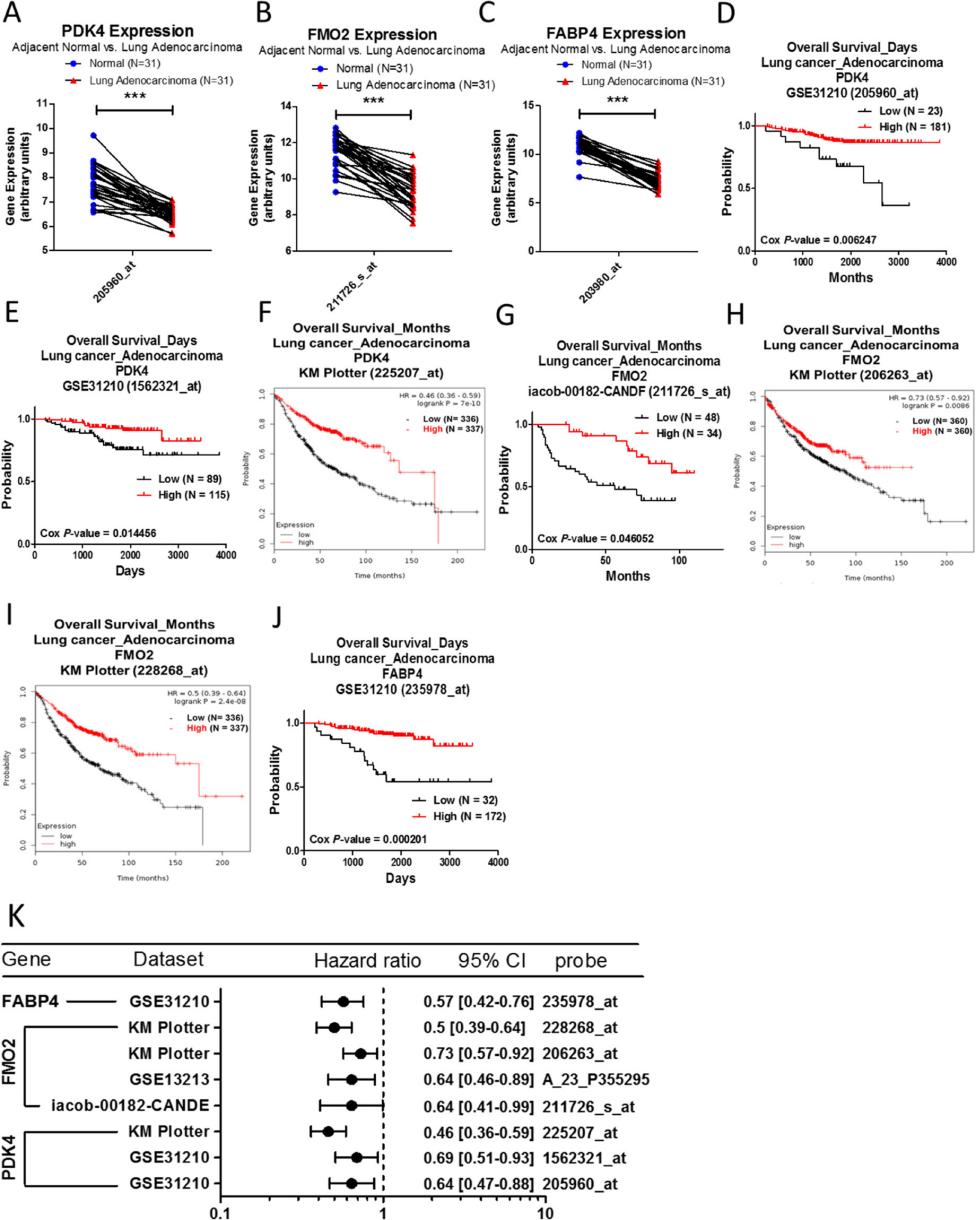
Analysis of PDK4, FMO2 and FABP4 in clinical lung adenocarcinoma patients using bioinformatics databases The gene expression of PDK4 (**A**), FMO2 (**B**), and FABP4 (**C**), comparing 31 pairs of clinical lung adenocarcinoma (red) and adjacent normal tissue (blue), was performed on GSE10072 microarray from the GEO database. The *p*-value of gene expression was calculated by *t*-test with Wilcoxon matched-pairs signed rank test. ^***^ represents *p* < 0.001. The survival curves comparing 2 populations with high (red) and low (black) gene expression in lung adenocarcinoma patients were performed on the PrognoScan database - PDK4 (**D**, **E**), FMO2 (**G**), and FABP4 (**J**), and the Kaplan–Meier plotter database – PDK4 (**F**) and FMO2 (**H**, **I**). The analysis criteria of the PrognoScan and Kaplan–Meier plotter databases were Cox *p*-value < 0.05 and log-rank *p*-value < 0.05 respectively. Raw data of the GEO and the PrognoScan databases were extracted and re-plotted by GraphPad Prism 5 software. (**K**) The forest plots showed hazard ratios (95% CI, confidence interval) identified from the PrognoScan and Kaplan–Meier plotter databases.

mRNA expression of PDK4, FMO2, and FABP4 between lung adenocarcinoma and normal lung tissue was analyzed by using the Oncomine database, and listed in Table 4. The expressions of PDK4, FMO2, and FABP4 are significantly downregulated in lung adenocarcinoma compared to normal lung tissue in several datasets, and this phenomenon was also observed in GSE10072 array (Figure 4A–4C). In cancer patients with lung adenocarcinoma, the survival curve analysis indicated that the population with higher expression of PDK4 (Figure 4D–4F), FMO2 (Figure 4G–4I), or FABP4 (Figure 4J) is correlated with better survival rates. The prognostic values of PDK4, FMO2, and FABP4 are shown in Figure 4K.

**Table 4:** Analysis of PDK4, FMO2 and FABP4 mRNA expression in lung adenocarcinoma compared to normal tissue from Oncomine database

Gene	Fold change (Cancer/Normal)	*P*-value	Gene Ranking (Top%)	Samples (Normal : Tumor)	Dataset	Probe
**PDK4**	−5.162	1.27E-24	1	20 : 226	Okayama	225207_at
	−9.834	2.27E-13	2	25 : 25	Wei	225207 _at
	−2.415	1.92E-16	4	49 : 58	Landi	205960_at
	−4.074	2.98E-7	5	30 : 27	Su	205960_at
	−4.33	1.56E-4	8	17 : 132	Bhattacharjee	36739_at
	−2.618	1.77E-4	9	19 : 20	Stearman	36739_at
	−4.593	6.29E-27	2	58 : 58	Selamat	ILMN_1684982
	−3.095	9.03E-7	7	10 : 86	Beer	U54617_at
	−10.368	2.77E-6	2	5 : 40	Garber	IMAGE:78946
**FMO2**	−3.77	4.98E-18	1	20 : 226	Okayama	211726_s_at
	−7.811	4.49E-15	2	25 : 25	Wei	228268_at
	−5.528	6.60E-28	1	49 : 58	Landi	211726_s_at
	−4.329	2.47E-10	2	30 : 27	Su	211726_s_at
	−8.705	1.62E-53	1	58 : 58	Selamat	ILMN_1732158
	−9.102	1.02E-24	1	65 : 65	Hou	228268_at
	−7.56	7.38E-6	2	5 : 39	Garber	IMAGE:80507
	−11.062	9.47E-19	1	10 : 86	Beer	Y09267_at
**FABP4**	−14.421	9.74E-28	1	20 : 226	Okayama	203980_at
	−20.112	1.42E-17	1	25 : 25	Wei	203980_at
	−19.625	1.89E-17	1	30 : 27	Su	203980_at
	−14.293	1.90E-37	1	49 : 58	Landi	203980_at
	−68.043	3.67E-13	1	17 : 132	Bhattacharjee	38430_at
	−13.918	2.07E-7	4	19 : 20	Stearman	38430_at
	−26.532	1.26E-12	2	10 : 86	Beer	J02874_at
	−9.214	1.34E-5	3	5 : 40	Garber	IMAGE:2308848
	−12.842	5.80E-44	1	58 : 58	Selamat	ILMN_1773006

### Analysis of CDKN2A, NDRG4, SFN, and PHLDA2 in lung adenocarcinoma

**Figure 5:**
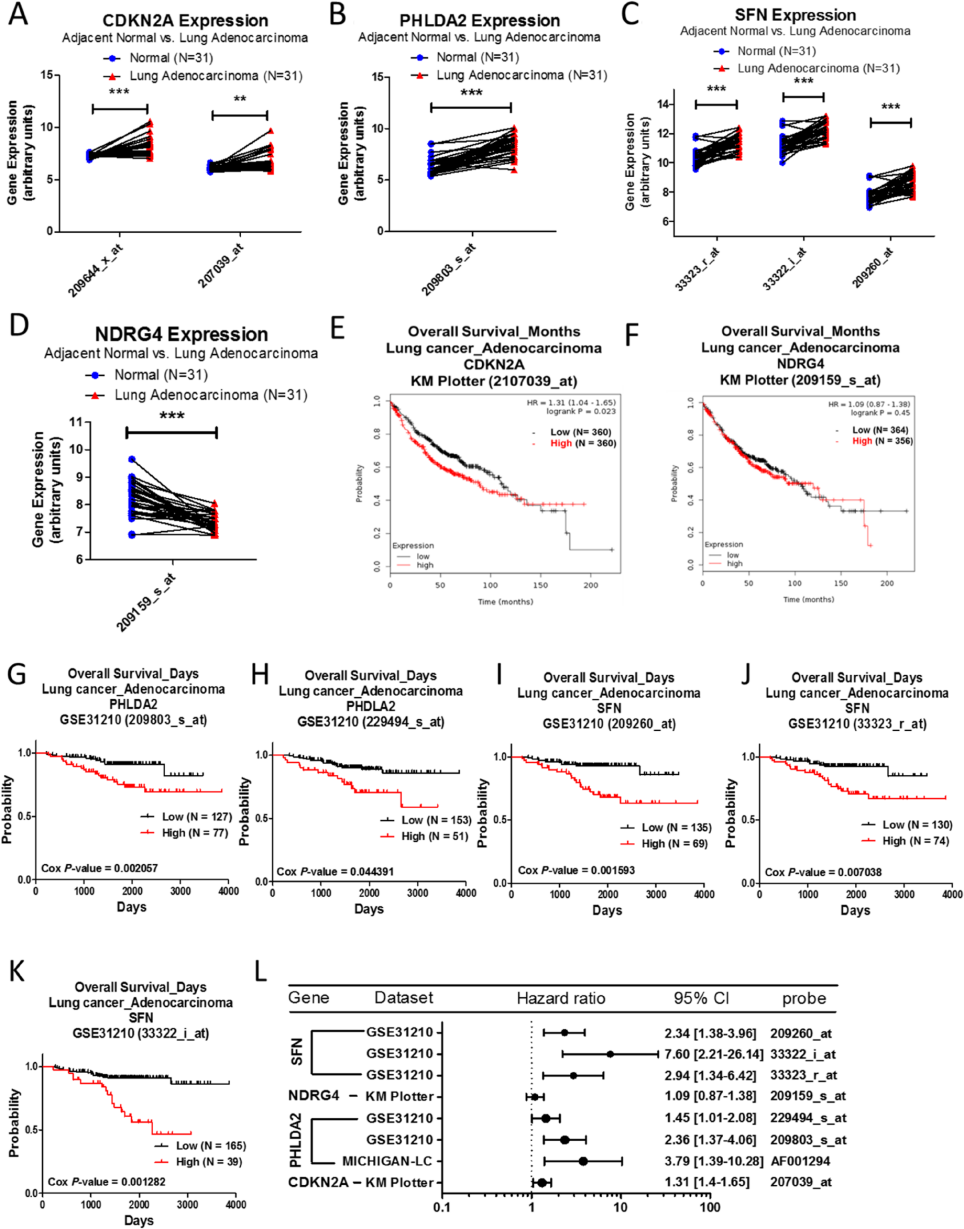
Analysis of CDKN2A, PHLDA2, SFN, and NDRG4 in clinical lung adenocarcinoma patients using bioinformatics databases The gene expression of CDKN2A (**A**), PHLDA2 (**B**), SFN (**C**), and NDRG4 (**D**), comparing 31 pairs of clinical lung adenocarcinoma (red) and adjacent normal tissue (blue), was performed on a GSE10072 microarray from the GEO database. The *p*-value of gene expression was calculated by *t*-test with Wilcoxon matched-pairs signed rank test. ^***^ represents *p* < 0.001, ^**^ represents *p* < 0.01. The survival curves comparing 2 populations with high (red) and low (black) gene expression in lung adenocarcinoma patients were performed on the Kaplan–Meier plotter database – CDKN2A (**E**) and NDRG4 (**F**), and PrognoScan database – PHLDA2 (**G**, **H**) and SFN (**I**–**K**). The analysis criteria of PrognoScan and Kaplan–Meier plotter databases were Cox *p*-value < 0.05 and log-rank *p*-value < 0.05 respectively. Raw data of the GEO and PrognoScan databases were extracted and re-plotted by GraphPad Prism 5 software. (**L**) The forest plots showed hazard ratios (95% CI, confidence interval) identified from the PrognoScan and Kaplan–Meier plotter databases.

We observed that the expression of CDKN2A, PHLDA2, and SFN are upregulated and the expression of NDRG4 is downregulated in lung adenocarcinoma when compared to normal lung tissue, which was also confirmed by the Oncomine database, listed in Table 5. In the GSE10072 array, expression levels of CDKN2A, PHLDA2, and SFN are significantly upregulated in lung adenocarcinoma compared to normal lung tissue in several datasets (Figure 5A–5C), and NDRG4 is downregulated (Figure 5D). The survival curve analysis showed that lung adenocarcinoma patients with high levels of CDKN2A expression are associated with poorer survival rates (Figure 5E). The expression level of NDRG4, however, has no significant effects on survival outcomes for patients with lung adenocarcinoma (Figure 5F). Higher expressions of PHLDA2 (Figure 5G, 5H), or SFN (Figure 5I–5K) are also associated with poorer survival. The forest plots showed the prognostic values of CDKN2A, PHLDA2, SFN, and NDRG4 (Figure 5L).

**Table 5:** Analysis of CDKN2A, PHLDA2, SFN, and NDRG4 mRNA expression in lung adenocarcinoma compared to normal tissue from Oncomine database

Gene	Fold change (Cancer/Normal)	*P*-value	Gene Ranking (Top%)	Samples (Normal : Tumor)	Dataset	Probe
**CDKN2A**	2.793	1.08E-8	1	20 : 226	Okayama	225207_at
	3.203	2.43E-6	2	25 : 25	Wei	225207 _at
	2.030	7.82E-11	4	49 : 58	Landi	205960_at
	3.161	4.32E-9	5	65 : 45	Hou	205960_at
	2.206	0.01	8	19 : 20	Stearman	36739_at
	3.506	7.68E-4	9	10 : 86	Beer	36739_at
**PHLDA2**	4.207	4.25E-9	1	25 : 25	Wei	211726_s_at
	5.73	1.80E-4	2	17 : 132	Bhattacharjee	228268_at
	4.027	1.99E-19	1	49 : 58	Landi	211726_s_at
	3.634	1.09E-7	2	30 : 27	Su	211726_s_at
	2.387	1.42E-17	1	58 : 58	Selamat	ILMN_1732158
	2.212	1.35E-5	1	65 : 45	Hou	228268_at
	4.015	9.11E-8	2	19 : 20	Stearman	IMAGE:80507
	2.799	2.05E-11	1	10 : 86	Beer	Y09267_at
**SFN**	4.721	1.98E-11	2	25 : 25	Wei	33323_r_at
	2.049	1.60E-12	5	49 : 58	Landi	33322_i_at
	6.459	3.04E-8	2	30 : 27	Su	209260_at
	2.410	4.06E-6	5	19 : 20	Stearman	33322_i_at
	4.487	1.67E-24	1	58 : 58	Selamat	ILMN_1806607
	2.302	2.74E-5	6	10 : 86	Beer	X57348_s_at
**NDRG4**	−3.519	1.78E-12	1	20 : 226	Okayama	203980_at
	−5.009	1.03E-13	1	25 : 25	Wei	203980_at
	−3.923	5.21E-9	1	30 : 27	Su	203980_at
	−4.402	2.32E-7	4	19 : 20	Stearman	38430_at
	−2.448	1.60E-14	2	65 : 45	Hou	J02874_at
	−2.152	4.63E-4	3	5 : 40	Garber	IMAGE:2308848
	−3.007	1.04E-18	1	58 : 58	Selamat	ILMN_1773006

### Analysis of AGR2, CLDN3, and AQP5 in lung adenocarcinoma

**Figure 6:**
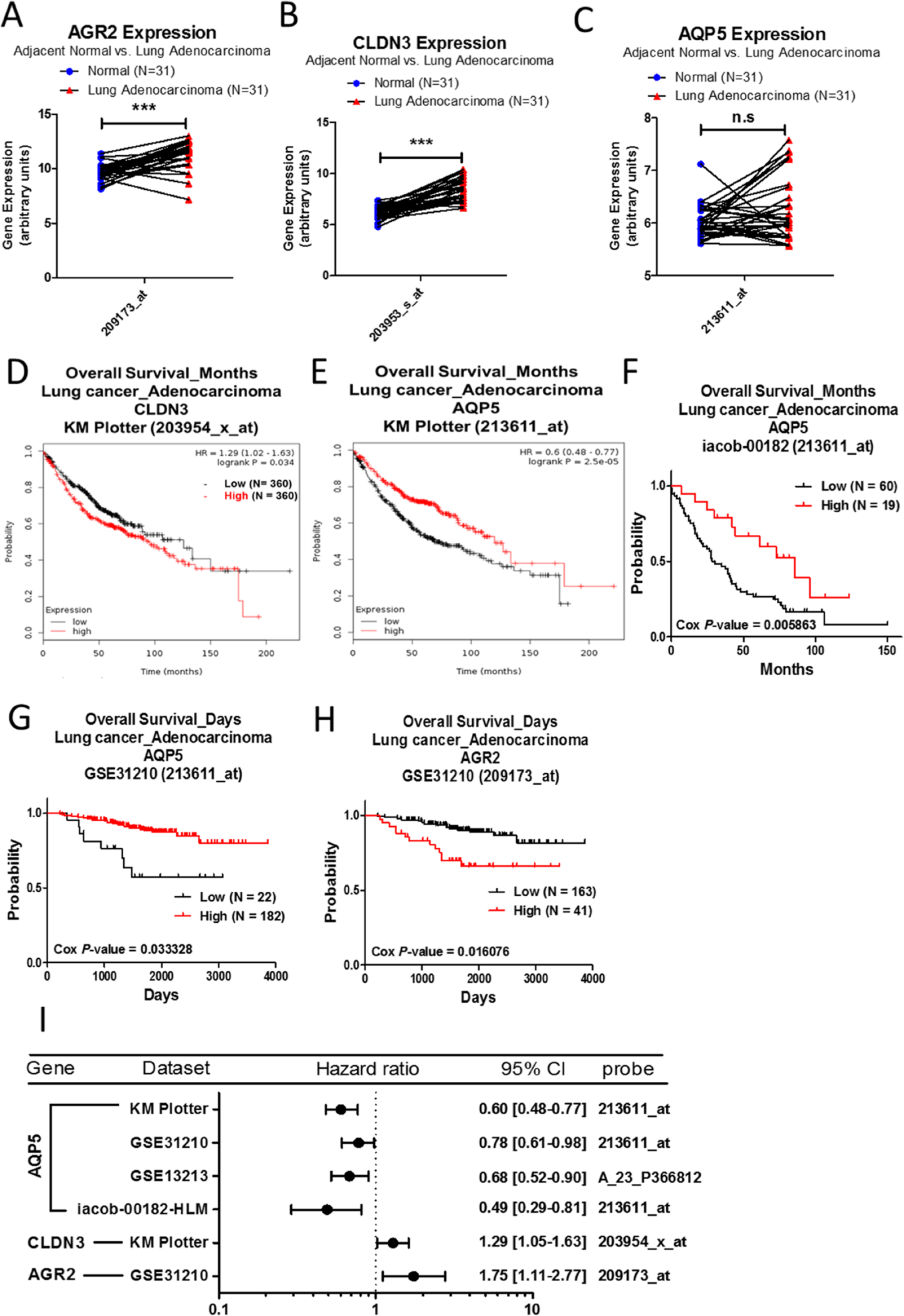
Analysis of AGR2, CLDN3, and AQP5 in clinical lung adenocarcinoma patients using bioinformatics databases The gene expression of AGR2 (**A**), CLDN3 (**B**), and AQP5 (**C**), comparing 31 pairs of clinical lung adenocarcinoma (red) and adjacent normal tissue (blue), was performed on a GSE10072 microarray from the GEO database. The *p*-value of gene expression was calculated by *t*-test with Wilcoxon matched-pairs signed rank test. ^***^ represents *p* < 0.001 and n.s. represents no significance. Survival curves comparing 2 populations with high (red) and low (black) gene expression in lung adenocarcinoma patients were performed on the Kaplan–Meier plotter – CLDN3 (**D**) and AQP5 (**E**), and PrognoScan databases – AQP5 (**F**, **G**) and AGR2 (**H**). The analysis criteria of the PrognoScan and Kaplan–Meier plotter databases were Cox *p*-value < 0.05 and log-rank *p*-value < 0.05 respectively. The raw data of the GEO and PrognoScan databases were extracted and re-plotted by GraphPad Prism 5 software. (**I**) The forest plots showed hazard ratios (95% CI, confidence interval) identified from the PrognoScan and Kaplan–Meier plotter databases.

Analysis of mRNA expression from the Oncomine database revealed that AGR2, AQP5, and CLDN3 are upregulated in lung adenocarcinoma, compared to normal lung tissue, and these results are listed in Table 6. In the GSE10072 array, we also observed that expression levels of AGR2 and CLDN3 are significantly upregulated in lung adenocarcinoma when compared to normal lung tissue (Figure 6A, 6B). However, the expression of AQP5 showed no significant change in the GSET10072 array (Figure 6C). The survival curve analysis showed that high expression of CLDN3 is correlated with poorer rates of survival in lung adenocarcinoma patients (Figure 6D). However, the population with a higher expression of AQP5 has better survival outcome (Figure 6E–6G). Higher expression of AGR2 is also associated with poorer survival rates (Figure 6H). The prognostic values of AGR2, CLDN3, and AQP5 were shown as forest plots (Figure 6I).

**Table 6:** Analysis of AGR2, AQP5, and CLDN3 mRNA expression in lung adenocarcinoma compared to normal tissue from Oncomine database

Gene	Fold change (Cancer/Normal)	*P*-value	Gene Ranking (Top%)	Samples (Normal : Tumor)	Dataset	Probe
**AGR2**	2.965	9.38E-11	6	20 : 226	Okayama	228969_at
	5.586	1.83E-15	1	25 : 25	Wei	209173 _at
	2.779	9.67E-11	7	49 : 58	Landi	209173_at
	3.434	1.49E-8	1	30 : 27	Su	209173_at
	2.902	0.003	3	17 : 132	Bhattacharjee	38827_at
	2.677	1.80E-5	6	19 : 20	Stearman	38827_at
	2.393	5.73E-11	7	58 : 58	Selamat	ILMN_1814151
**AQP5**	2.841	2.80E-6	10	25 : 25	Wei	213611_at
**CLDN3**	3.287	5.33E-12	4	20 : 226	Okayama	203954_x_at
	3.594	1.50E-11	1	25 : 25	Wei	203954_x_at
	3.282	1.71E-7	2	30 : 27	Su	203954_x_at
	2.193	4.34E-16	2	49 : 58	Landi	203954_x_at
	3.707	1.61E-8	1	19 : 20	Stearman	33904_at
	5.152	3.60E-5	1	17 : 132	Bhattacharjee	33904_at
	3.154	3.74E-14	4	58 : 58	Selamat	ILMN_1723042

### Analysis of IL33 in lung adenocarcinoma

**Figure 7:**
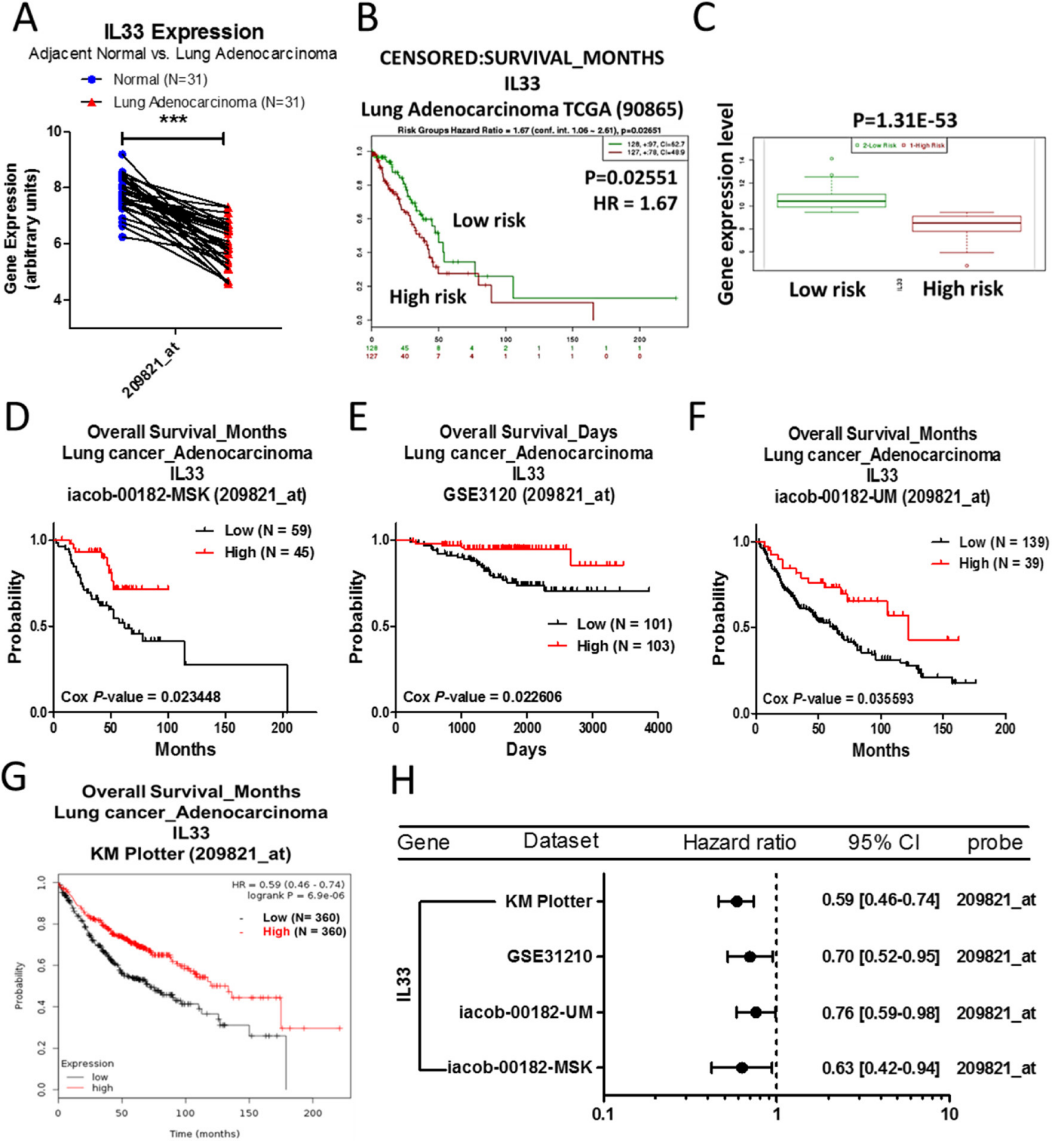
Analysis of IL33 in clinical lung adenocarcinoma patients using bioinformatics databases The gene expression of IL33 (**A**), comparing 31 pairs of clinical lung adenocarcinoma (red) and adjacent normal tissue (blue), was performed on a GSE10072 microarray from the GEO database. The *p*-value of gene expression was calculated by *t*-test with Wilcoxon matched-pairs signed rank test. ^***^ represents *p* < 0.001. (**B**) The survival curve was captured from the SurvExpress database, which divided lung adenocarcinoma patients into 2 populations of high (red) and low (green) risk, and (**C**) the box plots showed that the high risk (red) population has lower levels of IL33 expression, while the low risk (green) population has higher levels of IL33 expression. (**D**–**F**) The survival curves comparing 2 populations with high (red) and low (black) gene expression in lung adenocarcinoma patients were performed on the PrognoScan and (**G**) Kaplan–Meier plotter databases. The analysis criteria of the PrognoScan and Kaplan–Meier plotter databases were Cox *p*-value < 0.05 and log-rank *p*-value < 0.05 respectively. Raw data of the GEO and the PrognoScan databases were extracted and re-plotted by GraphPad Prism 5 software. (**H**) The forest plots showed hazard ratios (95% CI, confidence interval) identified from the PrognoScan and Kaplan–Meier plotter databases.

The mRNA expression of IL33 is significantly downregulated in lung adenocarcinoma compared to normal tissue (Table 7). We also found that IL33 expression is decreased in lung adenocarcinoma identified from the GSE10072 array (Figure 7A). The survival curve analysis performed using the SurvExpress database showed that the high risk population with lower expression of IL33 has poorer survival outcomes for lung adenocarcinoma patients (Figure 7B, 7C). This phenomenon was also observed in the PrognoScan (Figure 7D–7F) and Kaplan–Meier plotter databases (Figure 7G). The prognostic values of IL33 in lung adenocarcinoma was illustrated as a forest plot (Figure 7H).

**Table 7:** Analysis of IL33 mRNA expression in lung adenocarcinoma compared to normal tissue from Oncomine database

Gene	Fold change (Cancer/Normal)	*P*-value	Gene Ranking (Top%)	Samples (Normal : Tumor)	Dataset	Probe
**IL33**	−3.809	6.11E-20	1	20 : 226	Okayama	209821_at
	−4.325	1.04E-9	5	25 : 25	Wei	209821 _at
	−3.276	1.23E-21	2	49 : 58	Landi	209821_at
	−7.088	1.82E-9	3	30 : 27	Su	209821_at
	−3.582	2.02E-11	7	65 : 45	Hou	209821_at
	−2.163	8.36E-5	7	17 : 132	Bhattacharjee	35333_r_at
	−2.351	5.08E-5	8	19 : 20	Stearman	35333_r_at
	−4.258	5.82E-30	1	58 : 58	Selamat	ILMN_1809099

### Analysis of ZDHHC9, BTNL9, GNG11, and CPED1 in lung adenocarcinoma

**Figure 8:**
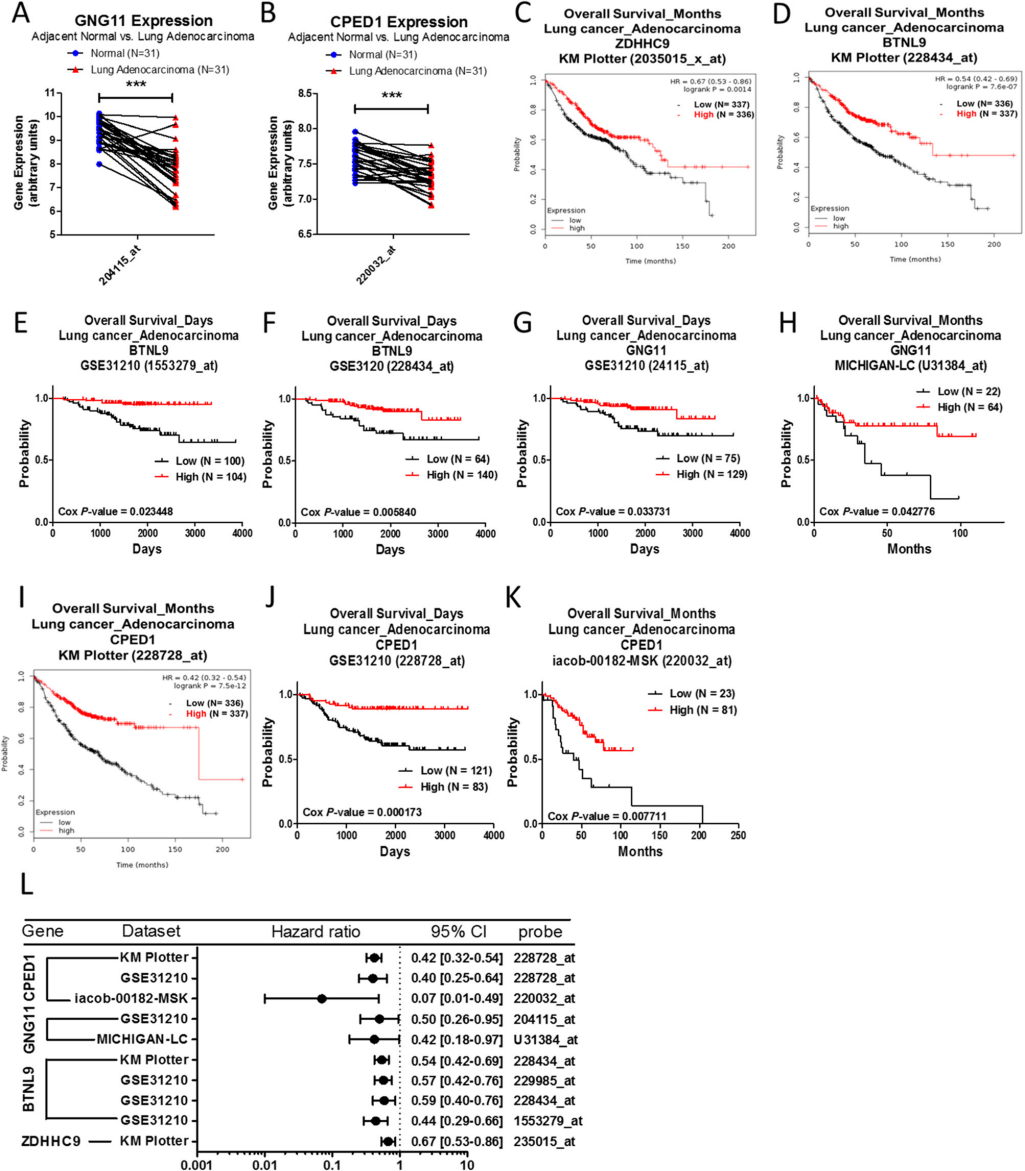
Analysis of ZDHHC9, BTNL9, GNG11 and CPED1 in clinical lung adenocarcinoma patients using bioinformatics databases The gene expression of GNG11 (**A**) and CPED1 (**B**), comparing 31 pairs of clinical lung adenocarcinoma (red) and adjacent normal tissue (blue), was performed on a GSE10072 microarray from the GEO database. The *p*-value of gene expression was calculated by *t*-test with Wilcoxon matched-pairs signed rank test. ^***^ represents *p* < 0.001. The survival curves comparing 2 populations with high (red) and low (black) gene expression in lung adenocarcinoma patients were performed on the Kaplan–Meier plotter database – ZDHHC9 (**C**), BTNL9 (**D**) and CPED1 (**I**), and the PrognoScan database – BTNL9 (**E**, **F**), GNG11 (**G**, **H**), and CPED1 (**J**, **K**). The analysis criteria of the PrognoScan and Kaplan–Meier plotter databases were Cox *p*-value < 0.05 and log-rank *p*-value < 0.05 respectively. Raw data of the GEO and PrognoScan databases were extracted and re-plotted by GraphPad Prism 5 software. (**L**) The forest plots showed hazard ratios (95% CI, confidence interval) identified from the PrognoScan and Kaplan–Meier plotter databases.

The mRNA expression of BTNL9, GNG11, or CPED1 is downregulated in lung adenocarcinoma when compared to normal tissue, and ZDHHC9 is upregulated (Table 8). In the GSE10072 array, GNG11 (Figure 8A) and CPED1 (Figure 8B) expression is downregulated in lung adenocarcinoma. There is no specific probe for ZDHHC9 and BTNL9 in the GSE10072 array. Survival curve analysis revealed that lung adenocarcinoma patients with high expression of ZDHHC9 (Figure 8C), BTNL9 (Figure 8D–8F), GNG11 (Figure 8G, 8H) or CPED1 (Figure 8I–8K) are correlated with better survival outcomes. The forest plot showed the prognostic values of ZDHHC9, BTNL9, GNG11, and CPED1 (Figure 8L).

**Table 8:** Analysis of ZDHHC9, BTNL9, GNG11 and CPED1 mRNA expression in lung adenocarcinoma compared to normal tissue from Oncomine database

Gene	Fold change (Cancer/Normal)	*P*-value	Gene Ranking (Top%)	Samples (Normal : Tumor)	Dataset	Probe
**ZDHHC9**	2.295	8.84E-12	5	20 : 226	Okayama	222451_s_at
	2.621	9.16E-10	3	25 : 25	Wei	222451_s_at
	2.377	3.27E-19	1	58 : 58	Selamat	ILMN_1803824
**BTNL9**	−12.000	3.77E-14	3	20 : 226	Okayama	228434_at
	−10.916	6.99E-26	1	25 : 25	Wei	228434_at
	−9.102	1.02E-24	1	65 : 45	Hou	228434_at
**GNG11**	−3.630	9.97E-21	1	20 : 226	Okayama	204115_at
	−4.337	6.55E-11	4	25 : 25	Wei	204115_at
	−3.463	1.49E-12	1	30 : 27	Su	204115_at
	−3.277	7.32E-23	2	49 : 58	Landi	204115_at
	−3.485	7.75E-11	1	19 : 20	Stearman	37908_at
	−20.213	2.51E-11	1	17 : 132	Bhattacharjee	37908_at
	−6.202	6.90E-25	2	58 : 58	Selamat	ILMN_1782419
	−2.830	4.15E-11	3	10 : 86	Beer	U31384_at
	−4.296	3.94E-4	4	5 : 5	Wachi	204115_at
**CPED1**	−2.181	7.18E-14	2	25 : 25	Wei	220032_at
	−3.108	1.13E-17	2	65 : 45	Hou	228728_at
	−3.325	1.03E-34	1	58 : 58	Selamat	ILMN_1677038

### Identification of genetic regulation in lung adenocarcinoma using next-generation sequencing

**Figure 9:**
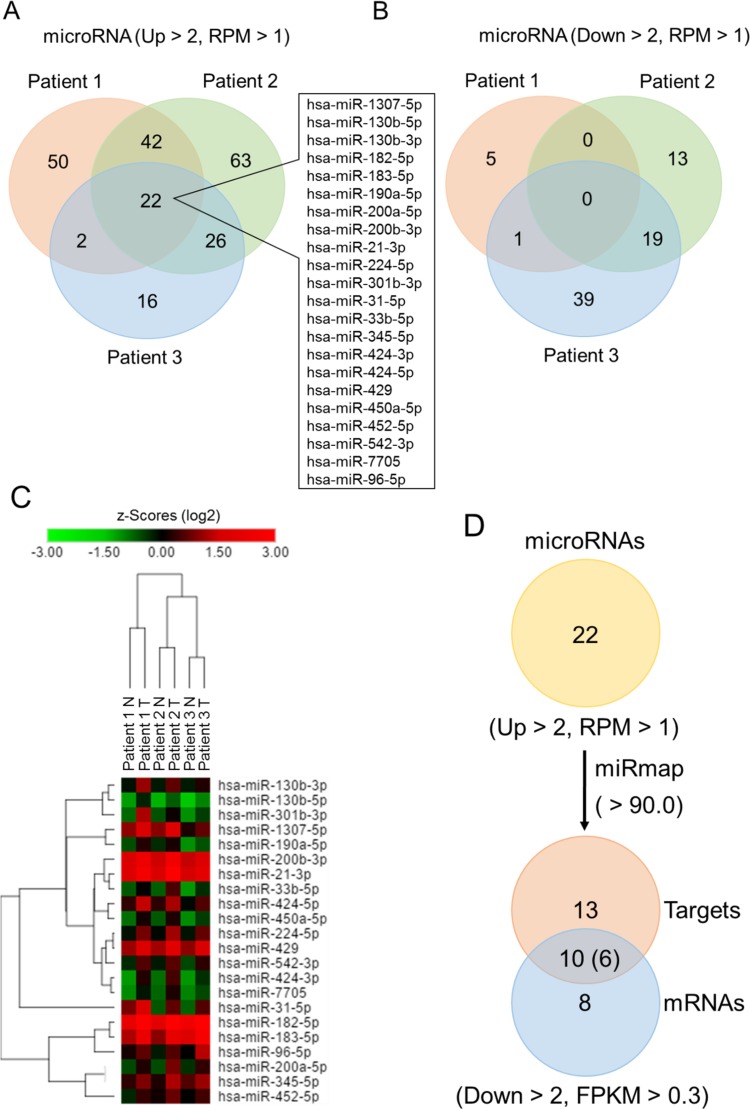
Identification of differentially expressed microRNAs in lung adenocarcinoma compared to adjacent normal tissue using next-generation sequencing Venn diagram analysis showed 22 upregulated microRNAs (**A**) and 0 downregulated microRNAs (**B**) in lung adenocarcinoma, compared to adjacent normal tissue from 3 pairs of clinical specimens. The criteria were fold change > 2 (tumor/normal) and reads per million (RPM) > 1. (**C**) The heatmap diagram showed the differentially expressed genes with z-score (log2) values by using color clustering on the GENE-E web-tool. Green represents downregulation (minimum = −3.0), and red represents upregulation (maximum = 3.0). (**D**) The “Targets” Venn diagram shows the predicted genes of microRNAs from the “microRNAs” Venn diagram using the miRmap web-site database. The selection threshold was miRmap score ≥ 90.0. The intersection Venn diagram between “mRNAs” and “Targets” showed total of 6 potential microRNA-mRNA interactions.

We simultaneously performed small RNA-seq in these 3 pairs of specimens using next-generation sequencing (Figure 9). We focused on microRNAs and found 22 upregulated microRNAs in lung adenocarcinoma using Venn diagram analysis (Figure 9A), which is listed in Table 9. The analysis criteria were fold change > 2 and reads per million (RPM) > 1. No microRNA with downregulated changes were found in our analysis (Figure 9B). Heatmap color clustering showed the expression patterns of each upregulated microRNA from these 3 pairs of specimens (Figure 9C). To further elucidate the genetic interactions in lung adenocarcinoma, we performed miRmap database for target prediction. Among 22 upregulated microRNAs, there were 13 putative targets, shown in the “Targets” Venn diagram (Figure 9D). The prediction threshold was miRmap score > 90.0. The Venn diagram analysis between 13 targets of microRNAs and 8 downregulated genes, shown in Figure 1B, indicates that there were 10 genetic interactions of microRNA-mRNA in lung adenocarcinoma (Figure 9D), which is listed in Table 10. Only 6 genes were involved in these 10 genetic interactions, due to the 3 genes have been attributed to different microRNAs.

**Table 9:** Differentially expressed microRNAs identified from next-generation sequencing data

miRNAs	Precursor	RPM (Reads per million)						T/N
		N1	T1	N2	T2	N3	T3	
hsa-miR-1307-5p	hsa-mir-1307	118.34	396.09	109.76	504.13	28.34	66.54	Up
hsa-miR-130b-5p	hsa-mir-130b	2.31	12.19	1.71	5.58	1.29	3.25	Up
hsa-miR-130b-3p	hsa-mir-130b	15.6	132.83	14.09	64.85	13.7	29.88	Up
hsa-miR-182-5p	hsa-mir-182	926.7	5350.27	731.46	5334.41	1779.57	15146.54	Up
hsa-miR-183-5p	hsa-mir-183	179.58	1197.91	111.57	917.47	471	2525.39	Up
hsa-miR-190a-5p	hsa-mir-190a	6.7	30.48	11	26.46	2.76	6.5	Up
hsa-miR-200a-5p	hsa-mir-200a	7.4	28.83	7.05	87.67	10.08	36.2	Up
hsa-miR-200b-3p	hsa-mir-200b	459.71	1338.61	275.25	2046.74	245.19	494.41	Up
hsa-miR-21-3p	hsa-mir-21	472.88	2282.29	448.1	7108.65	312.3	862.23	Up
hsa-miR-224-5p	hsa-mir-224	15.95	85.34	17.72	150.97	15.16	68.89	Up
hsa-miR-301b-3p	hsa-mir-301b	4.74	173.22	6.41	22.7	2.76	8.49	Up
hsa-miR-31-5p	hsa-mir-31	91.18	452.09	5.98	65.76	5.17	55.44	Up
hsa-miR-33b-5p	hsa-mir-33b	5.78	20.57	5.45	51.23	2.5	10.56	Up
hsa-miR-345-5p	hsa-mir-345	34.44	97.53	28.29	178.85	54.97	139.04	Up
hsa-miR-424-3p	hsa-mir-424	2.43	30.22	4.38	47.34	2.5	10.38	Up
hsa-miR-424-5p	hsa-mir-424	35.82	240.9	34.27	164.07	17.83	54.17	Up
hsa-miR-429	hsa-mir-429	143.41	407.01	153.53	673.9	119.92	359.34	Up
hsa-miR-450a-5p	hsa-mir-450a-1	4.39	25.53	5.87	28.4	2.93	8.67	Up
hsa-miR-450a-5p	hsa-mir-450a-2	4.39	25.4	5.87	28.27	2.93	8.67	Up
hsa-miR-452-5p	hsa-mir-452	11.79	36.95	23.81	72.5	20.16	45.05	Up
hsa-miR-542-3p	hsa-mir-542	11.33	54.23	13.99	60.05	7.41	34.67	Up
hsa-miR-7705	hsa-mir-7705	3	14.6	4.91	28.4	3.27	7.4	Up
hsa-miR-96-5p	hsa-mir-96	25.31	64	13.77	55.25	19.82	204.5	Up

**Table 10:** Genes selected between differentially expressed genes and putative targets of microRNA

miRNA	Predicted targets	miRmap score
hsa-miR-183-5p	BTNL9	92.2457
hsa-miR-200b-3p	CPED1	97.3127
hsa-miR-33b-5p	CPED1	91.5414
hsa-miR-429	CPED1	97.7498
hsa-miR-182-5p	FMO2	98.0884
hsa-miR-345-5p	FMO2	96.6797
hsa-miR-130b-5p	IL33	98.1906
hsa-miR-542-3p	IL33	96.593
hsa-miR-21-3p	NDRG4	95.067
hsa-miR-424-5p	PDK4	99.1986

## DISCUSSION

Lung cancer, one of the leading causes of cancer-related death worldwide [29], still has much that requires further study. In our project, we hoped to identify novel gene expression signature or genetic interactions of gene-microRNA in lung adenocarcinoma by using next-generation sequencing combined with systematic bioinformatics analysis.

We found 17 differentially expressed genes in lung adenocarcinoma compared to its adjacent normal lung tissue, which were classified into 6 functional groups based on a search of the literature. These results indicated that tumor progression is involved in alterations of various biological functions. We then summarized the potential oncogenic and tumor suppressor roles of these genes in lung adenocarcinoma (Supplementary Table 2).

TOX3 contains an HMG-box (high mobility group box) domain. The function of TOX3 remains unclear, but it may be involved in various DNA-dependent processes [30–32]. TOX3 polymorphisms and epigenetic regulation have been demonstrated in breast cancer [33] and lung cancer [34] respectively. In our study, TOX3 was significantly upregulated in lung adenocarcinoma, and higher expression of TOX3 is correlated with better survival outcome. We speculated that as more factors may be involved in TOX3-related mechanisms of tumor progression, more studies are needed to clarify the relationship between TOX3 expression and tumor progression.

SPDEF containing ETS domain has been reported to be overexpressed in many cancers [35–37]. Our study suggests that SPDEF may play an oncogenic role in lung adenocarcinoma, although some reports have shown that SPDEF can suppress cancer metastasis [38]. However, contrary effects of SPDEF on tumorigenesis require further research.

PDK4 is a mitochondrial protein that can regulate glucose metabolism through inhibition of pyruvate dehydrogenase complex. An aberrant metabolism is one of the characteristics of cancer cells. In liver cancer, PDK4 has been identified as a potential tumor suppressor [39]. Ironically, PDK4 exerts oncogenic effects in colon cancer [40]. In our study, PDK4 played a potential tumor suppressor role in lung adenocarcinoma.

FMO2 is an NADPH-dependent enzyme that catalyzes the oxygenation of substrates [41], but the effect of FMO2 on tumorigenesis is unclear. Although genetic polymorphisms of FMO genes may influence drug metabolism [42], we found that FMO2 might have tumor suppressor effects in lung adenocarcinoma.

FABP4 is involved in fatty acids trafficking and metabolism. Fatty acids serve as both an energy source and signaling molecules that can regulate various cellular functions [43]. The dysfunction of FABP proteins has been found to be associated with some metabolic diseases [44], and elevated FABP4 has been observed in many types of cancer [45–47]. Our data showed that FABP4 may have tumor suppressor effects in lung adenocarcinoma.

CDKN2A encodes two spliced transcripts, p16^INK4a^ and p14^ARF^, which regulate cell cycle progression through inhibition of CDK4 kinase and p53 respectively [48]. CDKN2A has been shown as a tumor suppressor in cancer progression [49], and its alterations, including epigenetic modifications, deletion, and mutations, frequently occur in cancers [50]. In our study, CDKN2A may have potential oncogenic effects in lung adenocarcinoma.

PHLDA2, located in an imprinted region on chromosome 11p15.5, has primarily been studied for its regulation of placental growth [51]. Although the role of PHLDA2 in cancer is unclear, our data showed that PHLDA2 may potentially exert oncogenic effects in lung adenocarcinoma.

SFN is involved in protein synthesis and epithelial cell growth. Numerous reports have demonstrated the molecular functions of SFN in keratinocytes and fibroblasts [52]. Furthermore, elevated expression of SFN has been reported in lung adenocarcinoma [53], and our study also found that SFN may exert oncogenic effects in lung adenocarcinoma.

NDRG4 is involved in the regulation of cell cycle progression [54] and has been identified as a novel tumor suppressor in colon cancer [55]. We found that NDRG4 levels were significantly decreased in lung adenocarcinoma, but with regard to survival analysis, the expression of NDRG4 has shown no significant influence on survival rates of lung cancer patients.

AGR2 is an endoplasmic reticulum (ER) protein which can catalyze protein folding. Its oncogenic role and increased expression have been reported in different types of cancer [56–58]. In our study, we found that AGR2 may serve as a potential prognostic biomarker of lung adenocarcinoma.

AQP5, aquaporin 5, is a water channel protein involved in pulmonary secretions, and elevated expression of AQP5 is associated with poor survival outcome in many types of cancer [59–61]. In our study, the expression of AQP5 was upregulated in lung adenocarcinoma, and its high expression correlated with better survival outcome.

CLDN3 regulates tight junctions of cell-cell adhesion in epithelial or endothelial cells and is overexpressed in ovarian [62] and colon cancer [63]. Loss of claudin 3 expression increases the metastatic ability of esophageal cancer [64], whereas claudin 3 is upregulated in lung adenocarcinoma [65]. Our study showed that claudin 3 may have oncogenic effects in lung adenocarcinoma.

IL33 is a cytokine involved in a spectrum of biological processes, and the chronic inflammatory signaling activation is known to be involved in cancer progression. The expression of IL33 in tumor tissues is depressed, but tumor stroma and serum have increased levels of IL33, suggesting the distinct functions of IL33 in cancer cells from the microenvironment [66]. IL-33 is shown to promote tumorigenesis and induce stemness in breast cancer [67]. In Apc^Min/+^ mice, epithelial-derived IL-33 can promote intestinal tumorigenesis [68]. These reports indicated the function of IL-33 in tumorigenesis is controversial. In our analysis, we found that IL33 may have tumor suppressor functions in lung adenocarcinoma.

ZDHHC9 is a palmitoyltransferase that can regulate palmitoylation of HRAS and NRAS. The function of ZDHHC9 in cancers is unclear, although inactivation of ZDHHC9 can reduce leukemogenic effects through repression of oncogenic NRAS [69]. According to our data, increased ZDHHC9 is observed in lung adenocarcinoma, but its high expression is correlated with better rates of survival.

BTNL9 belongs to the immunoglobulin superfamily, with the butyrophilin family modulating immune homeostasis [70]. Although the function of BTNL9 in tumorigenesis remains unclear, our results suggest that BTNL9 may serve as a tumor suppressor.

GNG11 is a lipid-anchored protein, which has been reported to inhibit cell growth [71] and regulate cellular senescence in lymphoma [72]. We found that GNG11 may exert suppressor functions in the tumorigenesis of lung adenocarcinoma.

CPED1, also known as C7orf58, contains a cadherin-like beta sandwich domain [73], but the molecular function of CPED1 is unclear. Our data showed that CPED1 may play a role as a potential tumor suppressor in lung adenocarcinoma.

**Figure 10:**
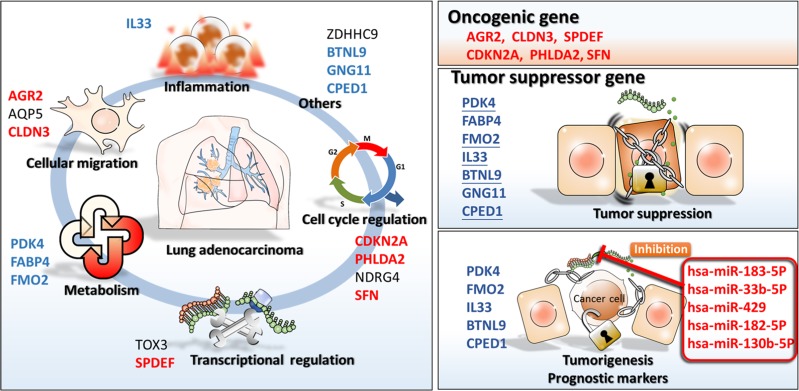
The proposed novel molecular signatures of gene regulations involved in lung adenocarcinoma

The summary of differentially expressed genes in the Oncomine database is shown in Supplementary Table 3. Seven of a total of 11 datasets showed similar patterns of genetic expression, suggesting that this molecular change is constant between lung adenocarcinoma and normal lung tissue. Thus, those genes found in this study may represent a novel gene expression signature in lung adenocarcinoma (Figure 10). The increased expression of AGR2 and decreased expression of IL33 have also been identified in other reports. We also analyzed the expression of microRNAs in lung adenocarcinoma (Table 9). Twenty-too upregulated microRNAs were identified in lung adenocarcinoma. We focused on microRNAs with predictable putative targets - BTNL9, FMO2, IL33, CPED1, and PDK4. Among these microRNAs, elevated expression of hsa-miR-183-5p [74], hsa-miR-33b-5p [75], hsa-miR-429 [76], hsa-miR-182-5p [77], and hsa-miR-130b-5p [78] have been associated with tumorigenesis in lung cancer. The function of hsa-miR-542-3p is unclear. However, since the genetic interactions of hsa-miR-183-5p-BTNL9, hsa-miR-33b-5p-CPED1, hsa-miR-429-CPED1, hsa-miR-182-5p-FMO2, hsa-miR-130b-5p-IL33, and hsa-miR-542-3p-IL33 have not been identified, these altered genetic regulations may play important roles in the progression of lung adenocarcinoma.

## MATERIALS AND METHODS

### Clinical lung adenocarcinoma specimens

Three pairs of tumors and adjacent non-tumor lung tissues were collected from the Division of Thoracic surgery and Division of Pulmonary and Critical Care Medicine, Kaohsiung Medical University Hospital (KMUH), Kaohsiung, Taiwan. Approval for these studies was obtained from the Institutional Review Board (IRB) of KMUH, and informed consent was obtained from all patients in accordance with the Declaration of Helsinki.

### Next-generation sequencing (NGS)

The expression profile of mRNA and microRNA was performed using NGS [22]. Three pairs of lung adenocarcinomas and adjacent normal specimens were used in this project. Total RNA was extracted by using Trizol® Reagent (Invitrogen, USA), according to the manufacturer's instructions. The cell lysates were applied to Welgene Biotechnology Company (Welgene, Taipei, Taiwan) for RNA-seq and small RNA-seq analysis. The criteria for differentially expressed mRNA analysis were fold change > 2 and fragments per kilobase million (FPKM) > 0.3. The criteria for differentially expressed microRNAs’ selection were fold change > 2 and reads per million (RPM) > 1.

### Oncomine database analysis

The Oncomine database contains over 18,000 microarray experiments and 35 major cancer types [23]. The raw data of mRNA expression in clinical lung adenocarcinoma and normal specimens (cancer vs. normal) were extracted from the Oncomine database (http://www.oncomine.org, Compendia biosciences, Ann Arbor, MI, USA). The criteria in the analysis were *P*-value < 1E-4, fold change > 2, and gene rank in top 10%. *P*-value was calculated using the Oncomine database through two-sided Student's *t*-test. For the comparison of genes in each dataset, raw data was extracted and re-plotted using the GENE-E web-tool (https://software.broadinstitute.org/GENE-E/), and the relative color scheme was used for clustering as minimum = 0 (blue) and maximum = 1 (yellow). Eleven datasets were selected for our analysis, including Hou (normal = 65 and lung adenocarcinoma = 45) [79], Landi (normal = 49 and lung adenocarcinoma = 58) [80], Selamat (normal = 58 and lung adenocarcinoma = 58) [81], Okayama (normal = 20 and lung adenocarcinoma = 226) [82], Su (normal = 30 and lung adenocarcinoma = 27) [83], Wei (normal = 25 and lung adenocarcinoma = 25)[84], Stearman (normal = 19 and lung adenocarcinoma = 20) [85], Bhattacharjee (normal = 17 and lung adenocarcinoma = 132) [86], Beer (normal = 10 and lung adenocarcinoma = 86) [87], Garber (normal = 5 and lung adenocarcinoma = 40) [88], and Wachi (normal = 5 and lung adenocarcinoma = 5) [89].

### SurvExpress database analysis

SurvExpress integrates the TCGA database (https://tcga-data.nci.nih.gov) which provides microarray information, including cancer type, survival, and gene expression. The correlation between IL33 mRNA expression and survival rate was analyzed on the SurvExpress web-databse (http://bioinformatica.mty.itesm.mx/SurvExpress). The dataset lung adenocarcinoma TCGA (*N* = 255) was used in our analysis. Samples of each dataset were split into 2 risk groups (high and low risk) of the same size, of which each group was determined by the ordered Prognostic Index (PI, high value for high risk) [27]. Prognostic Index (PI) is the linear component of the Cox model, computed by gene expression value multiplied with values estimated from the Cox fitting [90].

### PrognoScan database analysis

PrognoScan collects information of the GEO (Gene Expression Omnibus) microarray database, including cancer type, survival rates, and gene expression. The correlation between gene expression and overall survival rates was performed on the PrognoScan web-databse (http://www.abren.net/PrognoScan/) [25]. The raw data were extracted and re-plotted by GraphPad Prism 5 software (GraphPad Software, La Jolla, CA, USA). The threshold was determined as Cox *p*-value < 0.05. Samples of each dataset were divided into 2 expression groups (high and low) at the potential cutpoint. The cutpoint (from < 0.1 or > 0.9 quantile) was estimated by the minimum *P*-value approach [91], and the *P*-value correlation was calculated by the formula [92]. The hazard ratios (95% confidence intervals) of each dataset was calculated using the Cox proportional model, and are listed in the related Tables. HR = 0 represents no difference between 2 groups, HR < 1 represents better survival rate in the population with higher levels of expression, and HR > 1 represents better survival rates in the population with lower levels of expression. The specific probe of each dataset is listed in its related Figure.

### Kaplan–Meier plotter database analysis

The Kaplan-Meier plotter is a web-database providing the information on 54675 genes’ expression and survival rates in 10461 cancer samples, including 5143 breast, 1816 ovarian, 2437 lung, and 1065 gastric cancer patients [26]. The correlation of gene expression and overall survival rates in lung cancer was determined, and lung adenocarcinoma (*N* = 720) was selected in our analysis. Patients were split into 2 populations with the best cut-off, which was computed with median survival. The hazard ratios (95% confidence intervals) were calculated using the Cox proportional model, and are listed in the related Tables. The specific probe of each dataset was listed in its related Figure.

### Gene expression omnibus (GEO) database analysis

GEO is a web-database providing submitted high throughput gene expression data of microarrays, chips, or NGS (https://www.ncbi.nlm.nih.gov/geo/) [24]. We selected the microarray with accession number GSE10072, published in 2008 [80], for this project. This microarray provides gene expression information of 180 clinical lung adenocarcinoma and non-tumor samples. Here, we selected 31 pairs of lung adenocarcinoma with adjacent normal tissue for gene expression analysis. The raw data were analyzed and extracted from GEO2R (https://www.ncbi.nlm.nih.gov/geo/geo2r/), and re-plotted by GraphPad prism 5 software (GraphPad Software, La Jolla, CA, USA). The *p*-value was calculated by using *t*-test with Wilcoxon matched-pairs signed rank test.

### miRmap database analysis

miRmap is a web-tool database providing analysis of microRNA targets prediction (http://mirmap.ezlab.org/) [28]. It identifies the putative target genes by calculating the complementary ability of microRNA-mRNA interactions. The strength of mRNA repression is estimated for ranking potential candidate targets by employing various features, including thermodynamic, evolutionary, probabilistic or sequence-based features. The prediction results show a list of putative target genes with miRmap scores, which are a predictive reference values. Putative targets with miRmap scores ≥ 90.0 were selected for this project.

### Statistical analysis

The raw data extracted from GEO database were statistically analyzed using *t*-test with Wilcoxon matched-pairs signed rank test by GraphPad Prism 5 software (GraphPad Software, La Jolla, CA, USA).

## SUPPLEMENTARY MATERIALS TABLES


