# Correction: Identification of novel gene expression signature in lung adenocarcinoma by using next-generation sequencing data and bioinformatics analysis

**DOI:** 10.18632/oncotarget.26609

**Published:** 2019-01-15

**Authors:** Ya-Ling Hsu, Jen-Yu Hung, Yen-Lung Lee, Feng-Wei Chen, Kuo-Feng Chang, Wei-An Chang, Ying-Ming Tsai, Inn-Wen Chong, Po-Lin Kuo

**Affiliations:** ^1^ Graduate Institute of Medicine, College of Medicine, Kaohsiung Medical University, Kaohsiung, Taiwan; ^2^ School of Medicine, College of Medicine, Kaohsiung Medical University, Kaohsiung, Taiwan; ^3^ Division of Pulmonary and Critical Care Medicine, Department of Internal Medicine, Kaohsiung Medical University Hospital, Kaohsiung, Taiwan; ^4^ Division of Thoracic surgery, Department of Surgery, Kaohsiung Medical University Hospital, Kaohsiung, Taiwan; ^5^ Graduate Institute of Clinical Medicine, College of Medicine, Kaohsiung Medical University, Kaohsiung, Taiwan; ^6^ Welgene Biotech. Inc, Taipei, Taiwan; ^7^ Department of Respiratory Therapy, College of Medicine, Kaohsiung Medical University, Kaohsiung, Taiwan; ^8^ Center for Biomarkers and Biotech Drugs, Kaohsiung Medical University, Kaohsiung, Taiwan; ^9^ Institute of Medical Science and Technology, National Sun Yat-Sen University, Kaohsiung, Taiwan

**This article has been corrected:** The correct Acknowledgments and Funding information is given below:

**ACKNOWLEDGMENTS AND FUNDING**

This study was supported by grants from the Ministry of Science and Technology (MOST 106-2314-B-037-064; MOST 104-2320-B-037-014-MY3; MOST 104-2314-B-037-053-MY4; MOST 103-2320-B-037-006-MY3), the Kaohsiung Medical University “Aim for the Top 500 Universities Grant” (Grant No. KMU-TP105C05), the Kaohsiung Medical University “Aim for the Top Journals Grant” (Grant No. KMU-DT106005), and the Kaohsiung Medical University Hospital Research Foundation (KMUH106-6R13).

Original article: Oncotarget. 2017; 8:104831-104854. https://doi.org/10.18632/oncotarget.21022

