# Supplementary material for: Identification of novel gene expression signature in lung adenocarcinoma by using next-generation sequencing data and bioinformatics analysis
Source: Oncotarget. 2017 Sep 18;8(62):104831–54. doi: 10.18632/oncotarget.21022 (PMC5739604; doi:10.18632/oncotarget.21022)
Supplement: Supplementary file 1 [file oncotarget-08-104831-s001.pdf]

Identification of novel gene expression signature in lung adenocarcinoma by using next-generation sequencing data and bioinformatics analysis

SUPPLEMENTARY MATERIALS

Supplementary Table 1: Reference informations

1	Seksenyan A, Kadavallore A, Walts AE, de la Torre B, Berel D, Strom SP, Aliahmad P, Funari VA, Kaye J. TOX3 is expressed in mammary ER(+) epithelial cells and regulates ER target genes in luminal breast cancer. BMC cancer. 2015; 15:22.
2	Steffan JJ, Koul S, Meacham RB, Koul HK. The transcription factor SPDEF suppresses prostate tumor metastasis. The Journal of biological chemistry. 2016; 291:20826.
3	Zhao G, Jeoung NH, Burgess SC, Rosaaen-Stowe KA, Inagaki T, Latif S, Shelton JM, McAnally J, Bassel-Duby R, Harris RA, Richardson JA, Kliewer SA. Overexpression of pyruvate dehydrogenase kinase 4 in heart perturbs metabolism and exacerbates calcineurin-induced cardiomyopathy. American journal of physiology Heart and circulatory physiology. 2008; 294:H936–943.
4	Furuhashi M, Hotamisligil GS. Fatty acid-binding proteins: role in metabolic diseases and potential as drug targets. Nature reviews Drug discovery. 2008; 7:489–503.
5	Phillips IR, Shephard EA. Drug metabolism by flavin-containing monooxygenases of human and mouse. Expert opinion on drug metabolism & toxicology. 2017; 13:167–181.
6	Cashman JR, Zhang J. Human flavin-containing monooxygenases. Annual review of pharmacology and toxicology. 2006; 46:65–100.
7	Pal A, Potjer TP, Thomsen SK, Ng HJ, Barrett A, Scharfmann R, James TJ, Bishop DT, Karpe F, Godsland IF, Vasen HF, Newton-Bishop J, Pijl H, McCarthy MI, Gloyn AL. Loss-of-Function Mutations in the Cell-Cycle Control Gene CDKN2A Impact on Glucose Homeostasis in Humans. Diabetes. 2016; 65:527–533.
8	Wang X, Li G, Koul S, Ohki R, Maurer M, Borczuk A, Halmos B. PHLDA2 is a key oncogene-induced negative feedback inhibitor of EGFR/ErbB2 signaling via interference with AKT signaling. Oncotarget. 2015.
9	Arnold DR, Gaspar RC, da Rocha CV, Sangalli JR, de Bem THC, Correa CAP, Penteado JCT, Meirelles FV, Lopes FL. Nuclear transfer alters placental gene expression and associated histone modifications of the placental-specific imprinted gene pleckstrin homology-like domain, family A, member 2 (PHLDA2) in cattle. Reproduction, fertility, and development. 2017; 29:458–467.
10	Schilling SH, Hjelmeland AB, Radiloff DR, Liu IM, Wakeman TP, Fielhauer JR, Foster EH, Lathia JD, Rich JN, Wang XF, Datto MB. NDRG4 is required for cell cycle progression and survival in glioblastoma cells. The Journal of biological chemistry. 2009; 284:25160–25169.
11	Shiba-Ishii A, Kano J, Morishita Y, Sato Y, Minami Y, Noguchi M. High expression of stratifin is a universal abnormality during the course of malignant progression of early-stage lung adenocarcinoma. International journal of cancer. 2011; 129:2445–2453.
12	Wang Z, Hao Y, Lowe AW. The adenocarcinoma-associated antigen, AGR2, promotes tumor growth, cell migration, and cellular transformation. Cancer research. 2008; 68(2):492-497.
13	Chae YK, Woo J, Kim MJ, Kang SK, Kim MS, Lee J, Lee SK, Gong G, Kim YH, Soria JC, Jang SJ, Sidransky D, Moon C. Expression of aquaporin 5 (AQP5) promotes tumor invasion in human non small cell lung cancer. PloS one. 2008; 3:e2162.
14	Takehara M, Nishimura T, Mima S, Hoshino T, Mizushima T. Effect of claudin expression on paracellular permeability, migration and invasion of colonic cancer cells. Biological & pharmaceutical bulletin. 2009; 32:825-831.
15	Feng Y, Zhu Y, Luo G, Wang Z, Yu P, Zheng L. [Expression and clinical significance of IL-33 in patients with non-small cell lung cancer]. Xi bao yu fen zi mian yi xue za zhi = Chinese journal of cellular and molecular immunology. 2016; 32:808–811.
16	Liu P, Jiao B, Zhang R, Zhao H, Zhang C, Wu M, Li D, Zhao X, Qiu Q, Li J, Ren R. Palmitoylacyltransferase Zdhhc9 inactivation mitigates leukemogenic potential of oncogenic Nras. Leukemia. 2016; 30:1225–1228.
17	Arnett HA, Viney JL. Immune modulation by butyrophilins. Nature reviews Immunology. 2014; 14:559–569.
18	Hossain MN, Sakemura R, Fujii M, Ayusawa D. G-protein gamma subunit GNG11 strongly regulates cellular senescence. Biochemical and biophysical research communications. 2006; 351:645–650.
19	Zhu J and Chehab FF. Cutaneous Lipid Abnormalities In C7orf58 Knockout Mice Reveal A Critical Role For C7orf58 In Epidermal Lipid Homeostasis And The Late Steps Of Cholesterol Biosynthesis. Lipids: Regulation & Mechanism of Disease: Endocrine Society), pp. OR05-03-OR05-03. 2013.

Supplementary Table 2: Summary of predictive role of each gene in lung adenocarcinoma according to systematic bioinformatics analysis

Genes	Expression	Prognostic outcome	Potential role in tumorigenesis
TOX3	Up	Better	
AGR2	Up	Poor	Oncogene
SPDEF	Up	Poor	Oncogene
CDKN2A	Up	Poor	Oncogene
AQP5	Up	Better	
CLDN3	Up	Poor	Oncogene
SFN	Up	Poor	Oncogene
PHLDA2	Up	Poor	Oncogene
ZDHHC9	Up	Better	
PDK4	Down	Better	Tumor suppressor
FMO2	Down	Better	Tumor suppressor
NDRG4	Down		
CPED1	Down	Better	Tumor suppressor
GNG11	Down	Better	Tumor suppressor
IL33	Down	Better	Tumor suppressor
BTNL9	Down	Better	Tumor suppressor
FABP4	Down	Better	Tumor suppressor

Expression: UP and Down represents gene expression in lung adenocarcinoma compared to normal lung tissue.
Prognostic outcome represents the effects of high expression of each gene on survival.

Supplementary Table 3: Summary of differentially expressed genes in Oncomine database

	Datasets											
**Genes**	**Okayama**	**Landi**	**Su**	**Hou**	**Wei**	**Stearman**	**Selamat**	**Bhattacharjee**	**Beer**	**Wachi**	**Garber**	**11**
TOX3	Red	Red	Red	Red	Red	Red		Red				7
AGR2	Red	Red	Red	Red	Red	Red	Red	Red				8
SPDEF	Red		Red	Red	Red		Red					5
CDKN2A	Red	Red		Red	Red	Red			Red			6
AQP5					Red							1
CLDN3	Red	Red	Red	Red	Red	Red	Red	Red				8
SFN	Red	Red	Red		Red	Red	Red					6
PHLDA2		Red	Red	Red	Red	Red	Red	Red	Red			8
ZDHHC9	Red				Red		Red					3
PDK4	Green	Green	Green		Green	Green		Green	Green		Green	8
FMO2	Green	Green	Green	Green	Green		Green		Green		Green	8
NDRG4	Green		Green	Green	Green	Green	Green				Green	7
CPED1				Green	Green		Green					3
GNG11	Green	Green	Green		Green	Green	Green	Green		Green		8
IL33	Green	Green	Green	Green	Green	Green	Green	Green			Green	9
BTNL9	Green			Green	Green							3
FABP4	Green	Green	Green	Green	Green			Green	Green		Green	8
17	14	11	12	12	17	10	11	8	6	1	5	

Red and green represent up and down expression in each dataset.

Gray represents that the gene expression level is similar over half datasets (total = 11 dataset).

Yellow represents that the dataset shows the expression pattern with over half differentially expressed genes (total = 17 genes).
